# *Parkin* deficiency exacerbates fasting-induced skeletal muscle wasting in mice

**DOI:** 10.1038/s41531-022-00419-3

**Published:** 2022-11-17

**Authors:** Nesibe Peker, Mridula Sharma, Ravi Kambadur

**Affiliations:** 1 Koç University Research Center for Translational Medicine (KUTTAM), Koç University, Istanbul, 34010 Turkey; 2 School of Biological Sciences, Nanyang Technological University, 60 Nanyang Drive, 637551 Singapore; 3 Department of Biochemistry, School of Medicine, National University of Singapore, 8 Medical Drive, 117596 Singapore; 4 Cell and Molecular Biology Group, Singapore Institute for Clinical Sciences, 30 Medical Drive, 117609 Singapore

**Keywords:** Cell biology, Molecular biology, Gene regulatory networks

## Abstract

Parkinson’s Disease (PD) is a chronic and progressive neurodegenerative disease manifesting itself with tremors, muscle stiffness, bradykinesia, dementia, and depression. Mutations of mitochondrial E3 ligase, *PARKIN*, have been associated with juvenile PD. Previous studies have characterized muscle atrophy and motor deficits upon loss of functional *Parkin* in fly and rodent models. However, the mechanisms behind pathophysiology of *Parkin* deficient muscle remains to be elusive. Here, results suggested that knock down of *Parkin* significantly increases proteolytic activities in skeletal muscle cell line, the C2C12 myotubes. However, the atrogene levels increase moderately in *Parkin* deficient cell line. To further investigate the role of *Parkin* in skeletal muscle atrophy, *Parkin* knock out (KO) and wild type mice were subjected to 48 h starvation. After 48 h fasting, a greater reduction in skeletal muscle weights was observed in *Parkin* KO mice as compared to age matched wild type control, suggesting elevated proteolytic activity in the absence of *Parkin*. Subsequent microarray analyses revealed further enhanced expression of FOXO and ubiquitin pathway in fasted *Parkin* KO mice. Furthermore, a greater reduction in the expression of cytoskeleton genes was observed in *Parkin* KO mice following 48 h fasting. Collectively, these results suggest that *Parkin* deficiency exacerbates fasting-induced skeletal muscle wasting, through upregulating genes involved in catabolic activities in skeletal muscle.

## Introduction

Parkinson’s disease (PD) is the second most common neurodegenerative disease, characterised by tremors, muscle stiffness, impaired motor activities and dementia. Neuropathological analyses in PD brain have revealed selective and progressive loss of dopaminergic neurons in substantia nigra pars compacta (SNc)^1,2^. Besides, genetic analyses associated mutations of critical genes, including *PARKIN* (*PARK2*)^3^, *PINK1* (*PARK6*)^4^, DJ-1 (*PARK7*)^5^ and *LRRK2* (*PARK8*)^6^ with PD. Interestingly, studies documented neurodegeneration and parkinsonian phenotype, accompanied by impaired mitochondrial function upon loss of PINK1 and PARKIN in different models^7–11^. Further investigation revealed that these genes are involved in the regulation of mitochondrial biogenesis^12,13^, protein turnover^14^ and autophagic clearance^15^.

PINK1 and PARKIN cooperatively regulate mitochondrial turnover and selective clearance of mitochondria (mitophagy). Under physiological conditions, PINK1 (full length, 64 kDa) passes across outer mitochondrial membrane (OMM) and inner mitochondrial membrane (IMM) through TOM and TIM23 complexes, respectively. N-terminal of PINK1 that harbours mitochondrial targeting sequence (MTS) is cleaved by a peptidase called MPP. Also, PARL cleaves PINK1 within the transmembrane domain^16–18^. Upon depolarization of mitochondria, PINK1 accumulates on mitochondria as full-length form and becomes activated^19^. Active PINK1 autophosphorylates itself and phosphorylates PARKIN and ubiquitin at respective Ser65 residues^15,20–22^. Recent studies have shown that phosphorylation of ubiquitin at Ser65 further enhances PARKIN E3 ligase activity in a feed-forward mechanism^23^. These phosphorylation cascade leads to the recruitment of selective autophagy receptors and subsequent degradation of mitochondrial proteins^24,25^.

Indeed, *PARKIN* mutations are the most common cause of juvenile PD. In mouse model, loss of *Parkin* results in cognitive and behavioural defects^26^. Similarly, in fly model, *parkin* deficiency has been associated with behavioural abnormalities. Besides neuropathological signs, *Parkin* deficient models have impaired motor ability^27–29^. Strikingly, skeletal muscle mitochondria have been documented with abnormal cristae modelling^30^, hyperfusion, and dysfunction^28,31^, in *Parkin* mutant models.

Given that skeletal muscle mainly relies on oxidative phosphorylation (OXPHOS) for sustained muscle contraction, mitochondria are critical for maintaining skeletal muscle function^32^. As such, recent studies have reported atrophy of skeletal muscle upon loss of genes regulating mitochondrial turnover and mitophagy^33,34^. Loss of skeletal muscle mass and function, namely skeletal muscle atrophy, is a secondary symptom observed in chronic diseases, including cancer^35^, AIDS^36^, obesity^37^ and neurodegenerative diseases^38,39^. Metabolic perturbations, hyper catabolic activities, and loss of cytoskeleton proteins, in muscle cells, result in the loss of skeletal muscle integrity and mass. Skeletal muscle atrophy is mainly regulated through the activity of atrophy-related genes (atrogenes)^40^, under the control of FOXO transcription factors^41,42^. Indeed, FOXO transcription factors are also required for the control of atrogenes upon fasting and denervation in mouse model^43^.

Autophagy is a cellular clearance mechanism through which protein aggregates, long-lived proteins and dysfunctional organelles are degraded through lysosomes. Nutrient deprivation and several cellular stress factors have been shown to induce autophagy in mammalian systems. Mainly, autophagy is initiated by activation of ULK1 complex and class III PI3K complex I (PI3KC3 complex)^44^. PI3KC3 complex facilitates formation of phosphatidylinositol 3-phosphate (PI3P) at the omegasome, a special membrane structure that has been reported to be formed from ER^45^. Subsequent recruitment of PI3P-interacting proteins, zinc finger FYVE-type containing 1 (DFCP1) and WD repeat domain, phosphoinositide interacting proteins (WIPIs) leads to nucleation, thereby elongation of autophagosome^46,47^. Activation of ATG12~ATG5-ATG16L1 facilitates phagophore expansion through catalysing conjugation of ATG8/LC3 proteins to phosphatidylethanolamine (PE)^48,49^. Mature autophagosomes are transported to lysosomes through a system involving microtubules. Fusion with lysosome results in release of lysosomal acidic hydrolases into autophagosome and degradation of autophagic cargo. As impaired autophagy has been implicated in various diseases including cancer^50^, neurodegenerative diseases^51^, aging^52^ and obesity^53^. Autophagy has also been reported in muscle atrophy^54^. Also, loss of autophagy-related genes (ATGs) in mouse model has been shown to result in atrophy in mice^54^. FOXO transcription factors have been documented to regulate autophagy through controlling transcription of autophagy genes in skeletal muscle^55,56^.

Skeletal muscle atrophy in *Parkin* deficient in vitro and in vivo models has been documented by our group previously^31^. Also, *Parkin* has been reported to be required for training-induced adaptation in skeletal muscle^57^. In line with these findings, muscle-specific AAV-mediated overexpression of *Parkin* has been shown to ameliorate the atrophy phenotype observed in sarcopenic mice, through improving mitochondrial quality^58^, which further underscores the role of *Parkin* in skeletal muscle function.

Although compelling evidence suggests that *Parkin* is an important regulator of skeletal muscle homeostasis, mechanism behind this regulatory system remains to be investigated. In order to elucidate molecular mechanisms leading to atrophy phenotype in the presence of impaired mitochondria, catabolic activities upon loss of *Parkin* were investigated. Here, results showed that *Parkin* deficiency increases proteolytic activities in skeletal muscle cells. Results further revealed that loss of *Parkin* leads to a moderate increase in atrogene levels. Interestingly, phosphorylation and protein levels of FOXO1 were remarkably lower in *Parkin* KO mice as compared to wild type control. To delineate mechanisms regulating muscle atrophy in *Parkin* KO mice, mice were subjected to 48 h starvation and RNA-seq was performed using skeletal muscle. Strikingly, fasting-induced muscle wasting was exacerbated in *Parkin* KO mice subjected to 48 h fasting as compared to wild type controls. Collectively, these data suggest that loss of *Parkin* sensitises mice to fasting-induced muscle wasting through increased catabolic activities and reduced cytoskeleton gene expression.

## Results

### *Parkin* knockdown results in increased proteasomal activity in C2C12 cells

**Fig. 1:**
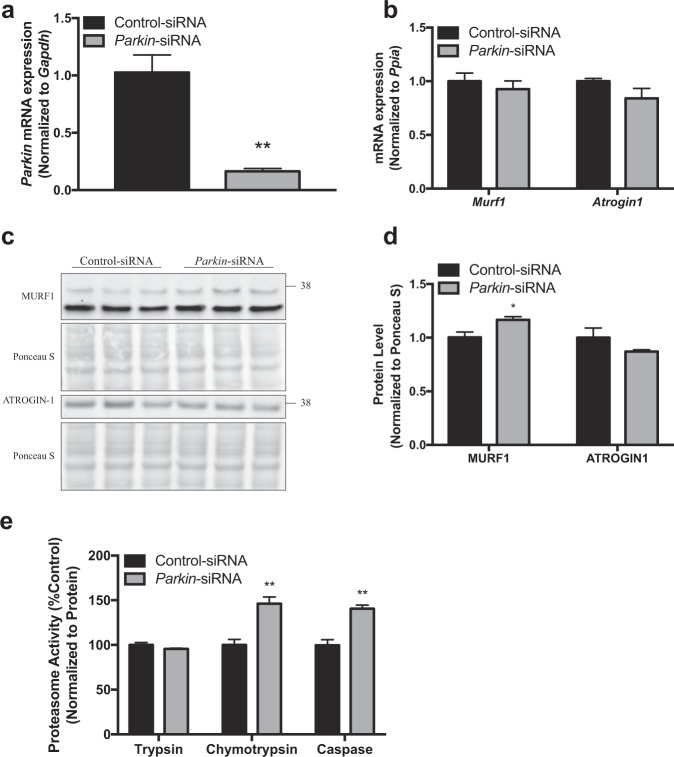
Parkin knock down increases 20S proteasomal activity in C2C12 myotube model. **a** Graph showing *Parkin* mRNA levels in non-targeting Control-siRNA and *Parkin*-siRNA transfected C2C12 myotubes. **b** Graph showing qPCR analysis of atrogenes *Murf1* and *Atrogin1* in Control-siRNA and *Parkin*-siRNA transfected myotubes. **c** Representative images of western blot analysis of MURF1 and ATROGIN-1 in Control-siRNA and *Parkin*-siRNA transfected myotubes. The levels of Ponceau-S were assessed as a loading control. **d** Graph showing quantification of protein levels of MURF1 and ATROGIN-1. **e** Graph showing three major 20S trypsin-like, chymotrypsin-like and caspase-like proteasomal activities in Control-siRNA and *Parkin*-siRNA transfected myotubes. Relative light units (RLU) were normalized to total protein and represented as percentage as compared to the control. Data represent mean±s.e.m. with error bars indicating s.e.m., n.s. not significant, *P < 0.05, **P < 0.01 (two-tailed Student’s t-test).

Previous study by our group has revealed skeletal muscle atrophy in *Parkin* KO mice as compared to wild type^31^, yet the mechanism behind the atrophy remained to be elusive. Hypercatabolism of skeletal muscle is commonly observed in various muscle wasting phenotypes and previous studies have documented enhanced proteasomal activity in sarcopenic and insulin resistant rodent models^59,60^. To explore whether lack of *Parkin* alters the levels of atrogenes and proteasomal activity, *Parkin* was knocked down in C2C12 myotubes using siRNA interference. Knock down efficiency of specific siRNA targeting *Parkin* was confirmed using qPCR analysis (Fig. 1a). Results revealed that knock down of *Parkin* does not alter the mRNA expression of *Atrogin1* and *Murf1* in C2C12 myotubes (Fig. 1b). Moreover, protein levels of MURF1 and ATROGIN1 were comparable with a subtle but significant increase of MURF1 levels in *Parkin* knock down myotubes as compared to control (Fig. 1c, d). Subsequent assessment of trypsin-like, chymotrypsin-like and caspase-like activity of 20S catalytic core using luminescence-based assay revealed increased chymotrypsin-like and caspase-like activities in *Parkin* knock down myotubes, whereas trypsin-like activity remained unaltered (Fig. 1e). Collectively, these data suggest that knock down of *Parkin* in skeletal muscle cells leads to activation of proteasomal activity and to some extent induction of MURF1 protein levels.

**Fig. 2:**
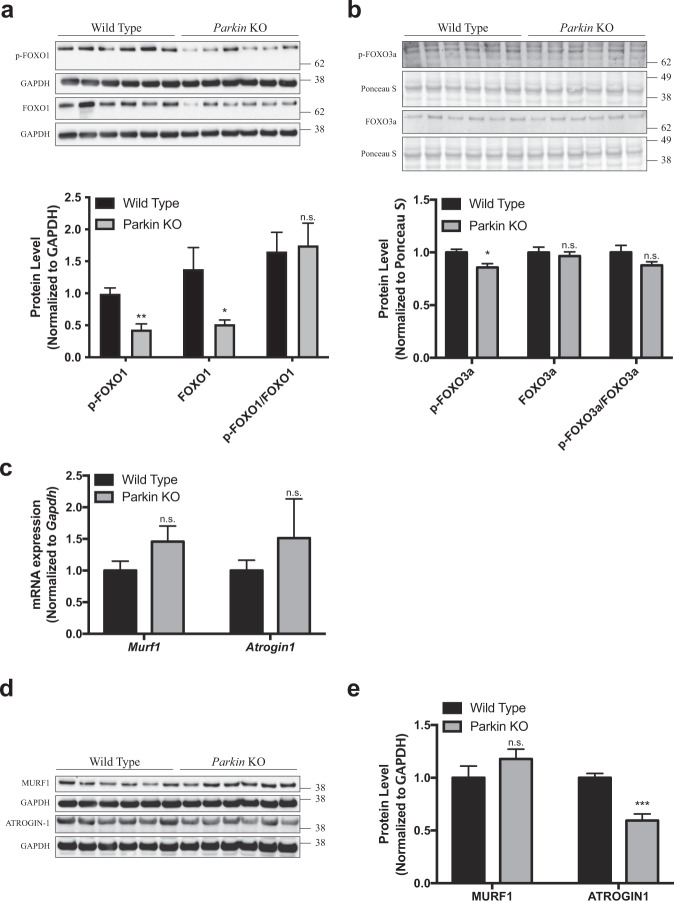
Knock out of Parkin results in moderate but not significant increase in MURF1 protein levels in Gas muscle. **a** Top, Representative images of western blot analysis of phosphorylated FOXO1 (p-FOXO1) and total FOXO1 in wild type and *Parkin* KO Gas muscle. The levels of GAPDH were assessed as a loading control (*n* = 6). Bottom, Graph showing quantification of p-FOXO1, total FOXO1 and p-FOXO1/total FOXO1 levels, normalized to GAPDH, in arbitrary units. (*n* = 6). **b** *Top*, Representative images of western blot analysis of phosphorylated FOXO3a (p-FOXO3a) and total FOXO3a in wild type and *Parkin* KO gas muscle. The levels of ponceau S were assessed as a loading control (*n* = 6). Bottom, Graph showing quantification of p-FOXO3a, total FOXO3a and p-FOXO3a/total FOXO3a levels, normalized to GAPDH, in arbitrary units. (*n* = 6). **c** Graph showing qPCR analysis of atrogenes *Murf1* and *Atrogin1* in wild type and *Parkin* KO gas muscle. **d** Representative images of western blot analysis of MURF1 and ATROGIN1 protein levels in wild type and *Parkin* KO gas muscle. The levels of GAPDH were assessed as a loading control (*n* = 6). **e** Graph showing quantification of MURF1 and ATROGIN1 protein levels, normalized to GAPDH, in arbitrary units (*n* = 6). Data represent mean ± s.e.m. with error bars indicating s.e.m., n.s. not significant, ***P < 0.001 (two-tailed Student’s *t*-test).

We next, investigated whether atrophy observed in *Parkin* KO mice resulted from the activation of AKT-FOXO pathway. Western blot analysis of phosphorylated FOXO1 (p-FOXO1) and total FOXO1 showed a significant decrease, although p-FOXO1/FOXO1 ratio remained unaltered in *Parkin* KO Gas muscle (Fig. 2a). Also, immunoblotting analysis revealed a significant reduction in protein levels of phosphorylated FOXO3a (p-FOXO3a) and FOXO3a protein levels, whereas p-FOXO3a/FOXO3a ratio was comparable between *Parkin* KO Gas muscle and wild type control (Fig. 2b). Importantly, a remarkable upregulation was observed in mRNA expression of atrogenes *Murf1* and *Atrogin1*, although the increase was not statistically significant (Fig. 2c). In agreement with in vitro observation, western blot analysis revealed a notable but not significant increase in MURF1 protein levels in *Parkin* KO Gas muscle (Fig. 2d, e) further suggesting that MURF1 may play a role in atrophy phenotype observed in *Parkin* KO mice, yet it may not be the main mechanism.

### Loss of *Parkin* results in differential gene expression in response to fasting in muscles

**Fig. 3:**
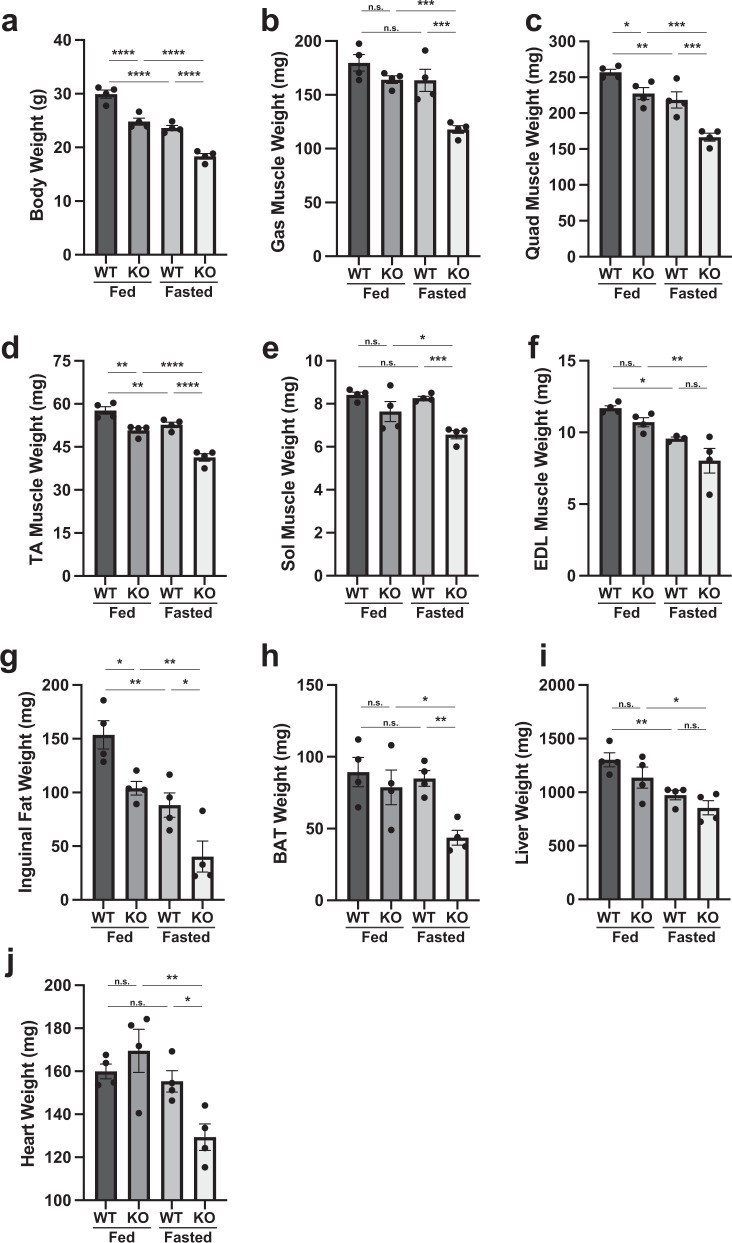
Loss of Parkin exacerbates the muscle and fat loss upon fasting in mouse model. **a** Graph showing body weights of wild type (WT) and *Parkin* KO (KO) as fed and following 48 h fasting (*n* = 4). **b**–**f** Graphs showing weights of Gas (**b**), Quad (**c**), TA (**d**), Sol (**e**) and EDL (**f**) in fed and fasted wild type (WT) and *Parkin* KO (KO) mice (*n* = 4). **g**, **h** Graphs representing the weights of inguinal fat pad tissue (**g**) and brown adipose tissue (BAT) (**h**) following 48 h fasting in wild type (WT) and *Parkin* KO (KO) mice as compared to fed counterparts (*n* = 4). **i** Graph showing liver weight following 48 h fasting in wild type (WT) and *Parkin* KO (KO) mice as compared to fed counterparts (*n* = 4). **j** Graph showing heart weight following 48 h fasting in wild type (WT) and *Parkin* KO (KO) mice as compared to fed counterparts (*n* = 4). Data represent mean ± s.e.m. with error bars indicating s.e.m., n.s. not significant, *P < 0.05, **P < 0.01, ***P < 0.001, ****P < 0.0001 (ordinary one-way ANOVA).

To investigate the main mechanism behind skeletal muscle atrophy observed in *Parkin* KO mice further, 12-week-old *Parkin* KO and age matched wild type mice were subjected to fasting for 48 h. Body weights of mice after 48 h fasting revealed a significant decrease in wild type and *Parkin* KO mice as compared to fed control (Fig. 3a). In addition to the body weight, hindlimb muscles were dissected and weights were recorded. Results revealed a remarkable muscle weight loss in all hindlimb muscles of fasted *Parkin* KO mice (Fig. 3b–f). Besides muscle, it is widely known that, intermittent fasting leads to a significant loss of adipose tissue deposits in mice^61^. Therefore, we next investigated whether fasting reduces fat deposits in *Parkin* KO mice. Consistent with reduced hindlimb muscle mass, results revealed a notable decrease in inguinal and brown fat deposits after starvation as compared to fed controls (Fig. 3g). Moreover, the reduction of BAT was significantly augmented in fasted *Parkin* KO mice as compared to fasted wild type mice (Fig. 3h). Although the weights of heart and liver have shown to be reduced, decrease in liver weights was comparable between fasted *Parkin* KO mice and wild type (Fig. 3i, j). Collectively, these results suggest that loss of *Parkin* exacerbates starvation-induced skeletal muscle and fat loss in mice.

**Fig. 4:**
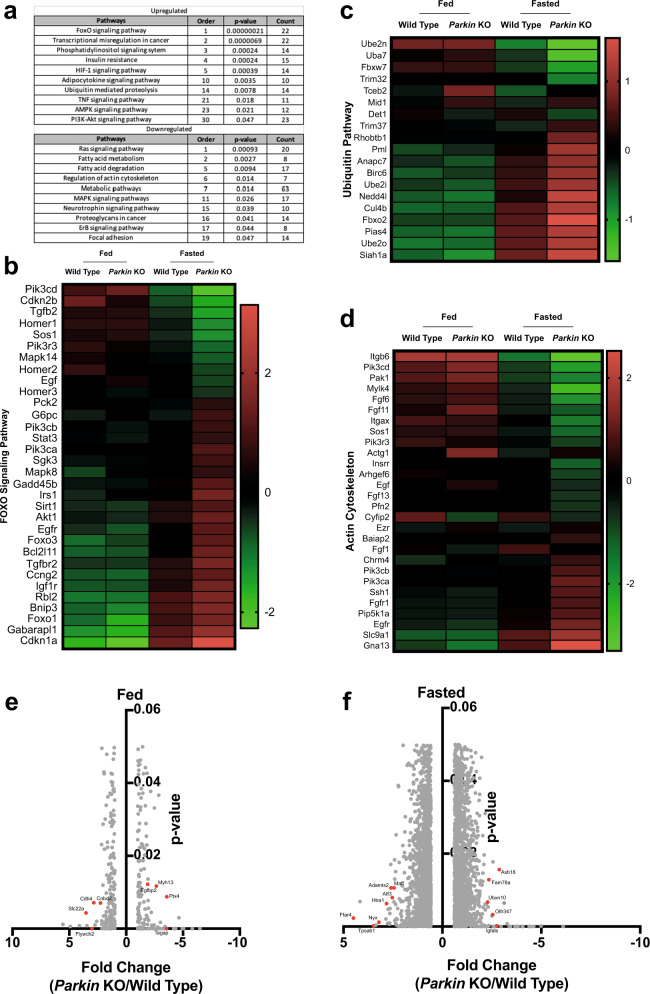
Microarray analysis reveals differentially regulated genes and pathways in Parkin KO mice upon fasting. **a** Signaling Pathways altered in significantly upregulated or downregulated genes obtained from hindlimb skeletal muscle of fed and fasted *Parkin* KO and wild type controls. **b** Heat map of microarray analysis showing the levels of differentially expressed genes involved in FOXO signalling pathway. Red and green indicate high and low gene expression respectively in log 2 base (*n* = 4). **c** Heat map of microarray analysis showing the levels of differentially expressed ubiquitin pathway genes. Red and green indicate high and low gene expression respectively in log 2 base (*n* = 4). **d** Heat map of microarray analysis showing the levels of differentially expressed actin cytoskeleton genes. Red and green indicate high and low gene expression respectively in log 2 base (*n* = 4). **e** Volcano plot showing differentially expressed genes (log2) in fed *Parkin* KO mice as compared to fed wild type mice with *p*-value on the *y*-axis (*n* = 4). **f** Volcano plot showing differentially expressed genes (log2) in fasted *Parkin* KO mice as compared to fasted wild type mice with *p*-value on the *y*-axis (*n* = 4).

To explore other mechanisms that may contribute to the atrophy phenotype observed in *Parkin* KO skeletal muscle, microarray was performed on total RNA isolated from Gas muscle. Expression of genes that are altered more than 1.5-fold or less than 0.66-fold with a 0.05 *p*-value cut-off in *Parkin* KO mice Gas muscle as compared to wild type Gas muscle was analysed using DAVID functional annotation tool. Of those 3234 genes, the expression of 1690 genes were significantly upregulated in *Parkin* KO Gas muscle as compared to wild type control whereas the expression of 1544 genes are reduced significantly in *Parkin* KO mice (Supplementary Data). Moreover, pathway analysis using DAVID online functional annotation tool revealed that *FOXO signalling pathway* is the most affected pathway with the lowest *p*-value (5.0E−8) indicating that FOXO pathway may have a role in the notable skeletal muscle loss observed in fasted *Parkin* KO mice (Fig. 4a). Furthermore, results suggest that fasting leads to a tendency towards increased levels of those genes in *Parkin* KO mice comparing to wild type controls which further underscores the role of FOXO signalling pathway in *Parkin* KO mice upon starvation (Fig. 4b).

The pathway analysis also revealed that fasting leads to a tendency towards increased levels of ubiquitin pathway genes as noted in both wild type and *Parkin* KO mice, although loss of *Parkin* does not result in a remarkable alteration in the levels of ubiquitin pathway genes per se (Fig. 4c). Strikingly, in agreement with the significant skeletal muscle loss, the upregulation of the expression of ubiquitin pathway genes observed upon fasting is augmented in *Parkin* KO mice, further suggesting that fasting enhances the activity of ubiquitin proteasome pathway, thereby may lead to a greater skeletal muscle loss in *Parkin* KO mice (Fig. 4c). Of those genes, the expression of *Rhobtb1*, *Pml*, *Anapc7*, *Birc6*, *Ube2i*, *Nedd4l*, *Cul4b*, *Fbxo2*, *Pias4*, *Ube2o* and *Siah1a* was found to be increased further in *Parkin* KO mice after 48 h fasting. In contrast, the expression of *Ube2n*, *Uba7*, *Fbxw7* and *Trim32* was found to be downregulated after 48 h fasting in both wild type and *Parkin* KO mice. Analysis also showed that the reduction in expression of these genes is significantly more in fasted *Parkin* KO mice as compared to that of fasted wild type mice (Fig. 4c).

Next, the expression levels of actin cytoskeleton genes was analysed. The analysis revealed that actin cytoskeleton genes tend to be downregulated after 48 h fasting and further decreased in *Parkin* KO mice which suggests that loss of *Parkin* exacerbates the degradation of skeletal muscle components, presumably through the activation of UPS (Fig. 4d).

To explore the top genes that are altered in *Parkin* KO mice comparing to wild type control, microarray analysis obtained from Gas muscle was analysed. Comparative analyses revealed that *Slc22a*, *Flywch2*, *Cdh4* and *Cndb2* are significantly upregulated top genes in *Parkin* KO mice as compared to wild type controls (Fig. 4e). In addition to genes that are upregulated, *Tagap*, *Ptx4*, *Myh13* and *Egfbp2* were downregulated dramatically in *Parkin* KO mice (Fig. 4e). When mice were fasted, results showed that *Ffar4*, *Tpsab1*, *Nyx*, *Htr1*, *Adamts2*, *Atf3* and *Maff* genes were the most significantly upregulated genes in the skeletal muscle of *Parkin* KO mice (Fig. 4f). Also, *Asb18*, *Igfals*, *Olfr347*, *Fam78a* and *Ubxn10* genes were reduced to a greater extent in *Parkin* KO mice most as compared to wild type controls (Fig. 4f). Collectively, these results reveal that loss of *Parkin* exacerbates fasting-induced skeletal muscle atrophy, through activating FOXO and ubiquitin pathway and reducing the expression of cytoskeleton genes as revealed by microarray analyses.

**Fig. 5:**
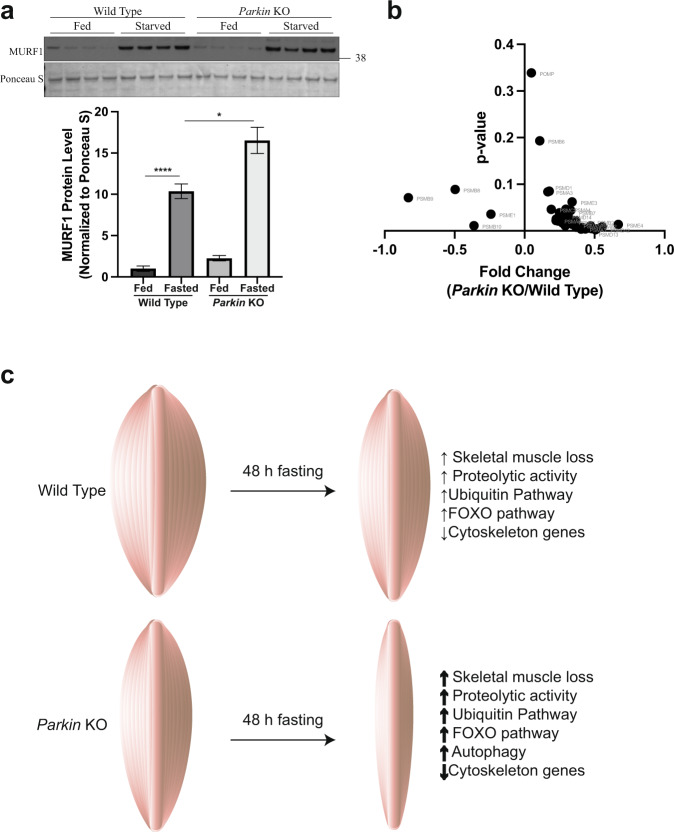
Parkin deficiency increases fasting-induced muscle mass loss. **a** Top, Representative images of western blot analysis of MURF1 in wild type and *Parkin* KO gas muscle. The levels of Ponceau S were assessed as a loading control (*n* = 4). *Bottom*, Graph showing quantification of MURF1 protein levels, normalized to Ponceau S, in arbitrary units (*n* = 4). **b** Volcano plot showing proteasome genes obtained from GSEA KEGG_Proteasome gene set and their expression levels (log2) in fasted *Parkin* KO mice as compared to fasted wild type mice with *p*-value on the *y*-axis (*n* = 4). **c** 48h fasting leads to muscle wasting in mice, which is further augmented in *Parkin* KO mice. Fasting-induced skeletal muscle wasting in *Parkin* KO mice is regulated through increased expression of genes associated with FOXO, ubiquitin pathway and autophagy, and decreased expression of cytoskeleton genes. Data represent mean±s.e.m. with error bars indicating s.e.m., *P < 0.05, ****P < 0.0001 (ordinary one-way ANOVA).

Next, to explore the skeletal muscle specific E3 ligase responsible for degradation of cytoskeleton proteins upon fasting, MURF1 protein levels were assessed. Western blot analysis revealed that upon fasting, MURF1 protein levels increase more in *Parkin* KO mice as compared to wild type control (Fig. 5a). Moreover, microarray analysis revealed that genes expressing for proteasome proteins were upregulated in *Parkin* KO mice following 48 h fasting (Fig. 5b), suggesting increased catabolism in skeletal muscle of *Parkin* KO mice following starvation.

Altogether, these data suggest that loss of *Parkin* in skeletal muscle results in an exacerbated muscle mass loss upon starvation. Microarray analyses indicate that increased expression of genes regulating proteasome activity, ubiquitin pathway and FOXO pathway may be responsible for degradation of skeletal muscle proteins in mice.

## Discussion

Skeletal muscle is a highly dynamic tissue with excessive and rapid energy demand which is mainly supplied by mitochondria. As such, mitochondrial quality control mechanisms have been of great interest for understanding the pathophysiology of skeletal muscle. Here, mechanism leading to skeletal muscle atrophy in the absence of *Parkin* was investigated. Results revealed that knock down of *Parkin* increased the catabolic activities in C2C12 cells, as assessed through proteolytic activity assays. Results also showed that *Parkin* deficiency increases the levels of MuRF1 protein moderately yet significantly, presumably through reducing FOXO1 and FOXO3 phosphorylation. Strikingly, in line with these findings, loss of *Parkin* exacerbates fasting-induced skeletal muscle wasting in mice.

It is widely known that food withdrawal results in increased activity of catabolic mechanisms, including activation of ubiquitin proteasome system (UPS) and autophagy^62,63^. Interestingly, during fasting, most tissues activate autophagy, yet this effect lasts a few hours. Exceptionally, only skeletal muscle keeps autophagy in an active state for days^64^. This indicates that increased catabolic activities perpetuate in skeletal muscle during fasting. Here, microarray analyses revealed that ubiquitin and FOXO pathway genes that are increased upon fasting are further increased in skeletal muscle of *Parkin* KO mice, underscoring the role of hypercatabolism in skeletal muscle upon loss of *Parkin*. In addition, reduced expression of cytoskeleton genes further suggests that *Parkin* deficiency sensitises skeletal muscle to fasting-induced wasting. Moreover, expressions of several critical autophagy genes are regulated under the control of FOXO transcription factors^56,65^. Especially, FOXO3 has been shown to be required for the induction of starvation-induced autophagy. However, atrogenes *Atrogin1* and *Murf1* were dispensable for FOXO3-driven autophagy activation, suggesting that two catabolic processes, autophagy and ubiquitin proteasome system, are independently controlled by FOXO3.

Autophagic clearance of mitochondria and subsequent remodelling of metabolic activities are crucial for skeletal muscle function. Impairment of mitochondria and respiratory complex subunits have been associated with several pathological conditions in skeletal muscle^32^. In addition, mitochondrial function and biogenesis were associated with muscle protein degradation^34^. Loss of *Parkin* has been shown to promote skeletal muscle atrophy in 4-week-old mice^31^. In this study, 12-week-old mice were fasted for 48 h to induce atrophy in skeletal muscle. Previous studies have reported protective role of *Parkin* in mouse skeletal muscle^66,67^. For example, mitochondrial function and biogenesis were altered in skeletal muscle of *Parkin* KO mice^31,66^.

Parkin ensures healthy pool of mitochondria through regulating autophagic clearance of mitochondria^19,68,69^. As such, function of Parkin mainly is controlled by canonical autophagy mechanism. Indeed, a recent study has shown that upon mitochondrial stress, phosphorylation of Parkin by ULK1 and AMPK, proteins responsible for initiation of autophagy upon cellular stress and nutrient deprivation, occurs before phosphorylation by PINK1^70^. This underlines how tightly Parkin is regulated by canonical autophagy pathway.

Autophagy is a critical contributing factor in maintenance of muscle mass and function in mice^71^. Studies have shown that loss of critical autophagy genes result in atrophy in skeletal muscle^54,72,73^. As such, increased expression of genes regulating autophagy may be a contributing factor in the loss of muscle mass in *Parkin* KO mice upon starvation, which requires further investigation. Indeed, autophagic flux has been reported to be elevated in skeletal muscle of *Parkin* KO mice. Gouspillou et al. showed that expression of genes controlling autophagic flux, such as *Map1lc3b*, *Gabarapl1* and *Bnip3* were increased in skeletal muscle of *Parkin* KO mice^66^. In line with this, microarray analysis revealed that levels of *Map1lc3b* (0.9 in fed comparison vs 1.6 fold in fasted comparison), *Gabarapl1* (0.8 in fed comparison vs 1.8 fold in fasted comparison) and *Bnip3* (0.8 in fed comparison vs 2.0 fold in fasted comparison) are significantly increased in *Parkin* KO mice as compared to wild type control upon starvation. Moreover, expression of genes regulating initiation of autophagy, *Ulk1* (1.1 in fed comparison vs 1.9 fold in fasted comparison) and *Wipi2* (0.9 in fed comparison vs 1.7 fold in fasted comparison) is upregulated in *Parkin* KO mice following fasting. Altogether, these suggest that expression of autophagy genes is increased in *Parkin* KO mice after fasting, which in turn may result in enhancement of catabolic activities in skeletal muscle.

Although mitochondrial quality is an important factor for maintaining homeostasis of skeletal muscle cells, skeletal muscle wasting eventually requires activation of catabolic activities to reduce muscle mass. Muscle specific E3 ligases target cytoskeleton proteins under denervation^40,74^ and starvation^75,76^ conditions. On the other hand, the role of E3 ligases in aged skeletal muscle remains elusive^77^. In this study, a slight, yet significant increase in MURF1 protein levels may be responsible for the exacerbated atrophy phenotype observed in *Parkin* KO mice upon fasting. Microarray analysis revealed differentially expressed genes in *Parkin* KO mice and wild type control in both fed and fasting conditions. These analyses may provide insight into the ubiquitin proteasome system regulators, especially E3 ligases targeting cytoskeleton proteins, and facilitate the investigation of molecular mechanisms dysregulated in skeletal muscle of *Parkin* KO mice.

Especially, further increase in genes expressing for proteins involved in catabolic activities, including proteasome subunits and FOXO pathway, and decrease in cytoskeleton genes upon fasting in *Parkin* KO mice suggest that future research should focus on the role of cellular quality control systems, such as autophagy and UPS in skeletal muscle of *Parkin* KO models.

## Methods

### Cell culture and transfection

C2C12 mouse myoblasts were purchased from ATCC (Manassas, VA) and were maintained in proliferation media supplemented with 10% fetal bovine serum (FBS; GIBCO, Carlsbad, CA) and 1% penicillin-streptomycin (P/S; GIBCO). Myoblasts were differentiated into myotubes by replacing proliferation media with differentiation media which is supplemented with 2% horse serum (HS; GIBCO) and 1% P/S. Fully differentiated myotubes were transfected with either non-targeting (Control-siRNA) or *Parkin*-specific (*Parkin*-siRNA) SMARTpool siRNA solutions as per manufacturer’s instructions (Dharmacon RNAi Tech, Lafayette, CO).

### RNA extraction and qPCR analysis

RNA was isolated using TRIzol reagent (Thermo Fisher Scientific, Waltham, MA, USA) according to manufacturer’s instructions. 1 µg RNA was converted into cDNA using the iScript cDNA cynthesis kit purchased from Bio-Rad (Hercules, CA). Gene expression was assessed using the SsoFast EvaGreen Supermix and CFX96 touch real-time PCR detection system as per manufacturer’s protocol (Bio-Rad).

### Protein extraction and western blot analysis

To extract total cell lysate from cells, cells were rinsed with PBS twice and lysed using RIPA buffer (1% NP-40, 0.1% SDS, 0.5% Na deoxycholate, 50 mM NaF, 0.2 mM Na_3_VO_4_) complemented with protease inhibitor cocktail (Sigma-Aldrich, St. Louis, MO), PhosSTOP (Sigma-Aldrich), and 1 mM PMSF (Sigma-Aldrich) by triturating cells 25 times with a 26-gauge needle assembled to a 1 ml syringe. To extract total cell lysate from tissues, tissues were mechanically lysed using Qiagen Tissue Lyser-II (Qiagen, Valencia, CA) for 3 times, 2 min each, at 30 Hz. Samples were centrifuged at 10,000 × *g* for 10 min at 4 °C. Supernatant containing total cell lysate was transferred into fresh tubes and protein concentration was estimated using Bradford assay as per manufacturer’s instructions (Bio-Rad). Equal amount of protein was resolved on 4–12% bis-tris precast gels (Invitrogen, Carlsbad, CA) and transferred onto nitrocellulose membrane (Bio-Rad) using either the XCell II Blot module wet transfer system or the iBlot 2 dry blotting system (Invitrogen) according to manufacturer’s instructions. Membranes were blocked in 5% milk solution for 1 h at room temperature prior to primary antibody hybridization for overnight at 4 °C. Antibodies used in this study are as follows; ATROGIN1 (1:500, CST, PAB15627), MURF1 (1:500, Regeneron Pharmaceuticals), p-FOXO1 (1:1000, CST, 9461), FOXO1 (1:1000, CST, 2880), p-FOXO3 (1:1000, CST, 9466), FOXO3 (1:500, CST, 7497), GAPDH (1:10,000, Santa Cruz, sc-32233), anti-mouse HRP (1:5000, Bio-Rad, 1706516), anti-rabbit HRP (1:5000, Bio-Rad, 1706515), anti-goat HRP (1:2000, Bio-Rad, 1721034). Membrane was washed 3 times 5 min each before and after secondary antibody hybridization which was performed at room temperature for 1 h. Protein levels were visualized using Western Lightning Plus-ECL (Perkin Elmer, Waltham, MA) and ChemiDoc Touch imaging instrument (Bio-Rad) or autoradiography (X-ray films). Protein levels were analysed using Image Lab software (Bio-Rad) and represented as bar graphs. All blots were processed in parallel and derive from the same experiment within the same experimental group.

#### Animal studies

*Parkin* knock out (*Park2*^tm1Shn^) mice with C57BL/6 background and wild type C57BL/6 mice were obtained from The Jackson Laboratory (Sacramento, CA, USA) and Biological Resource Centre (BRC, Singapore) respectively. Mice were housed in the Nanyang Technological University (NTU) Animal House Facility (Singapore), in light (12 h light/dark) and temperature (20–22 °C) controlled rooms. Animal experiments were approved by the Institutional Animal Ethics Committee (IACUC, Singapore) and performed by researchers with Responsible Care and Use of Laboratory Animal Course (RCULAC, Singapore) certificate. For starvation, 12-week-old wild type and *Parkin* KO mice were starved for 48 h. Control group was fed until 4 h before the dissection. Weights of heart, liver and total body were recorded. Mice were sacrificed at the same time and *M. gastrocnemius* (Gas), *M. Quadriceps* (Quad), *M. tibialis anterior* (TA), *M. soleus* (Sol) and *M. extensor digitorum longus* (EDL) hind limb muscles were dissected and weighed following the dissection. Brown adipose tissue and inguinal fat was collected from the interscapular region of the torso and posterior subcutaneous depots respectively.

### RNA extraction and microarray analysis

To determine differentially expressed genes in *Parkin* KO Gas muscle as compared to wild type control, total RNA was isolated from 12-week-old *Parkin* KO and age-matched wild type gas muscle using mirVANA™ miRNA isolation kit as per manufacturer’s guidelines (Thermo Fisher Scientific). RNA integrity was assessed using Agilent RNA 6000 Nano Kit (Agilent Technologies, Santa Clara, CA, USA). Subsequent microarray analyses were performed by Molecular Genomics (Singapore) using Agilent SurePrint G3 Custom GE 8 × 60 K, 1 colour platform as following: 100 ng/µl RNA was probed with Low Input AMP labelling Kit, One Colour (Agilent p/n 5190-2305) as per manufacturer’s instruction (One-colour microarray-based gene expression, analysis low input quick amp labelling, version 6.5). 100 ng/µl RNA was converted into cDNA using oligo-dt primers containing T7 RNA polymerase recognition site. Cyanine 3-CTP labelled cRNA was transcribed using T7 RNA polymerase. 600 ng cRNA was hybridized onto Agilent SurePrint G3 Human GE 8x60K microarray at 65 °C, 10xrpm for 17 hours in Agilent hybridization oven. In vitro transcription of Pathway analysis was performed with significantly upregulated (>1.5 fold) and downregulated (<0.66 fold) genes with a p-value cut off <0.05 using Kyoto Encyclopedia of Genes and Genomes (KEGG) pathway database integrated in the Database for Annotation, Visualization and Integrated Discovery (DAVID) tool. Protein class analysis and identification of significantly upregulated genes was carried out using Protein Analysis Through Evolutionary Relationships (PANTHER) gene list analysis software.

### Assessment of 20S proteasome activity

To determine 20S proteasome activity, C2C12 cells were seeded into clear bottom 96 well black plates at the density of 15,000 cells per cm^2^ and maintained in 100 µl The Proteasome-Glo™ cell-based reagent. Specific peptide substrate Succinyl-leucine-leucine-valine-tyrosine-aminoluciferin (Suc-LLVY-aminoluciferin), Z-leucine-arginine- arginine-aminoluciferin (Z-LRR-aminoluciferin) and Z-norleucine-proline-norleucine-aspartate-aminoluciferin (Z-nLPnLD-aminoluciferin) was added into the wells for the assessment of chymotrypsin-like, trypsin-like and caspase-like activities, respectively. 96-well plate was shaken on a plate shaker for 2 min at 700 rpm and incubated at room temperature for 10 min. Plate was read by Proteasome-Glo programme that is pre-installed by manufacturer using GloMax®96 microplate luminometer (Promega, Madison, WI, USA). Relative light units (RLU) detected by the machine were normalized to total protein and represented as percentage of control-siRNA transfected values.

### Statistical analyses

All experiments were performed at least three times, independently. In microarray analyses, significance analysis is performed with *T*-test and Benjamini-Horchberg false discovery rate (FDR; I-value) and fold change analysis was done by *T*-test. Two-tailed Student’s t-test and ordinary one-way ANOVA are applied where applicable.

### Reporting summary

Further information on research design is available in the Nature Research Reporting Summary linked to this article.

## Supplementary information

Unprocessed western blot images

Supplementary Data

Reporting Summary

## Data Availability

Unprocessed western blot images were provided in Supplementary Material. Microarray data corresponding to Fed and Starved is available as Supplementary Data, and also at the ArrayExpress Archive of Functional Genomics Data, with the ArrayExpress accession number E-MTAB-12273. The other data that support the findings of this study are available from the corresponding author upon reasonable request.

## Code availability

This study does not include any custom developed coding, as data processing was performed using online software using Kyoto Encyclopedia of Genes and Genomes (KEGG) pathway database integrated in the Database for Annotation, Visualization and Integrated Discovery (DAVID) tool.
