# Supplementary material for: Parkin deficiency exacerbates fasting-induced skeletal muscle wasting in mice
Source: NPJ Parkinsons Dis. 2022 Nov 17;8:159. doi: 10.1038/s41531-022-00419-3 (PMC9672397; doi:10.1038/s41531-022-00419-3)

Supplementary Figure 1

Uncropped western blots	
**Figure**	**Supplementary Figure**
Figure 1c	Supplementary Figure 1a
Figure 2a	Supplementary Figure 1b
Figure 2b	Supplementary Figure 1c
Figure 2d	Supplementary Figure 2a
Figure 5a	Supplementary Figure 2b

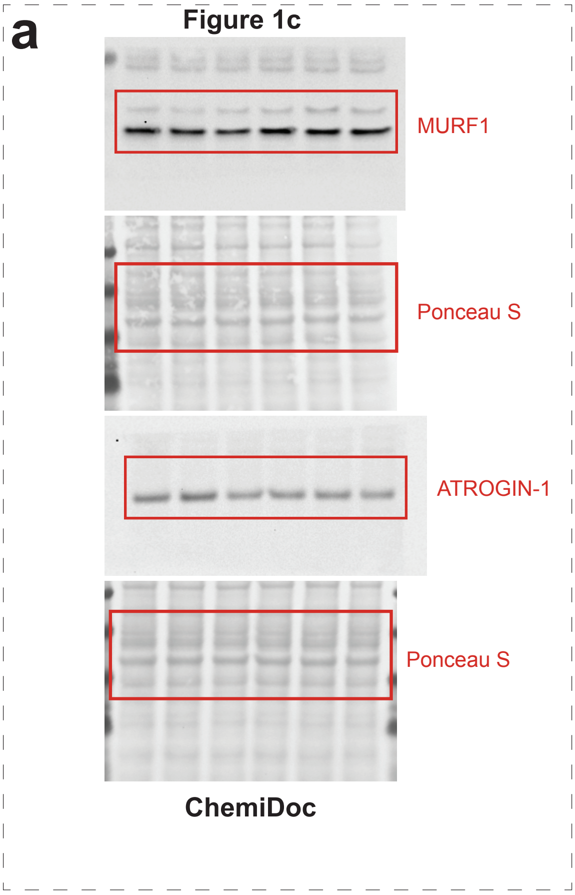

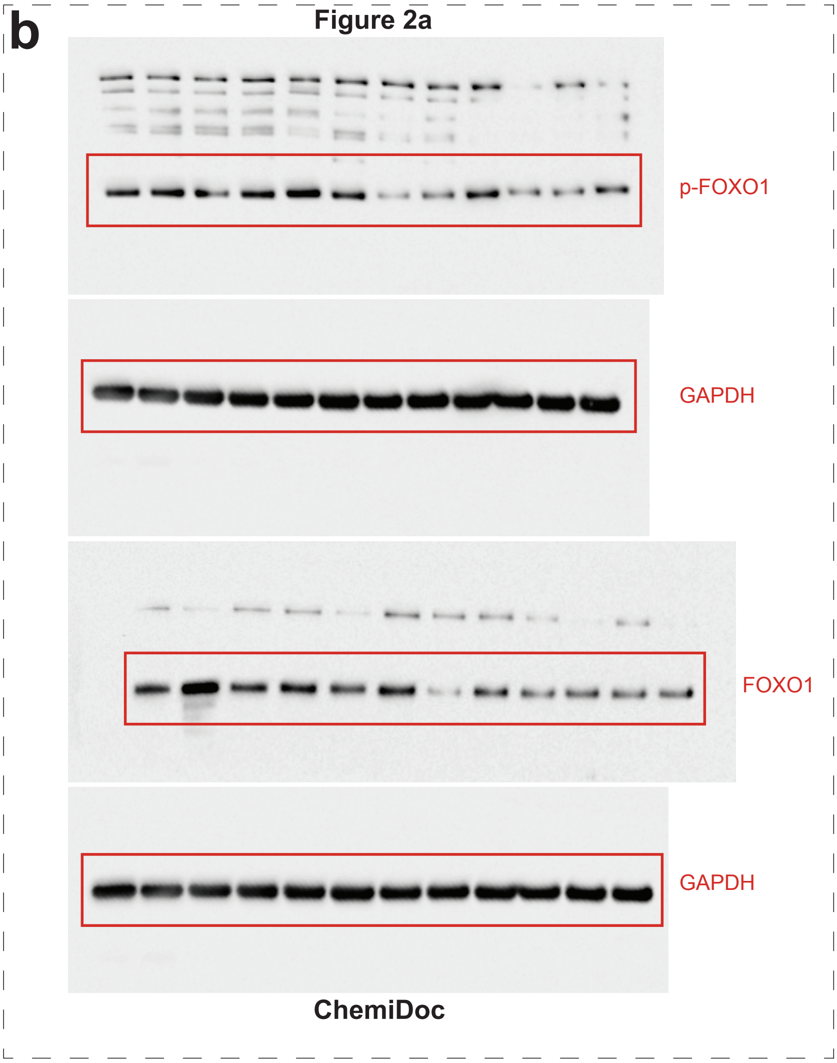

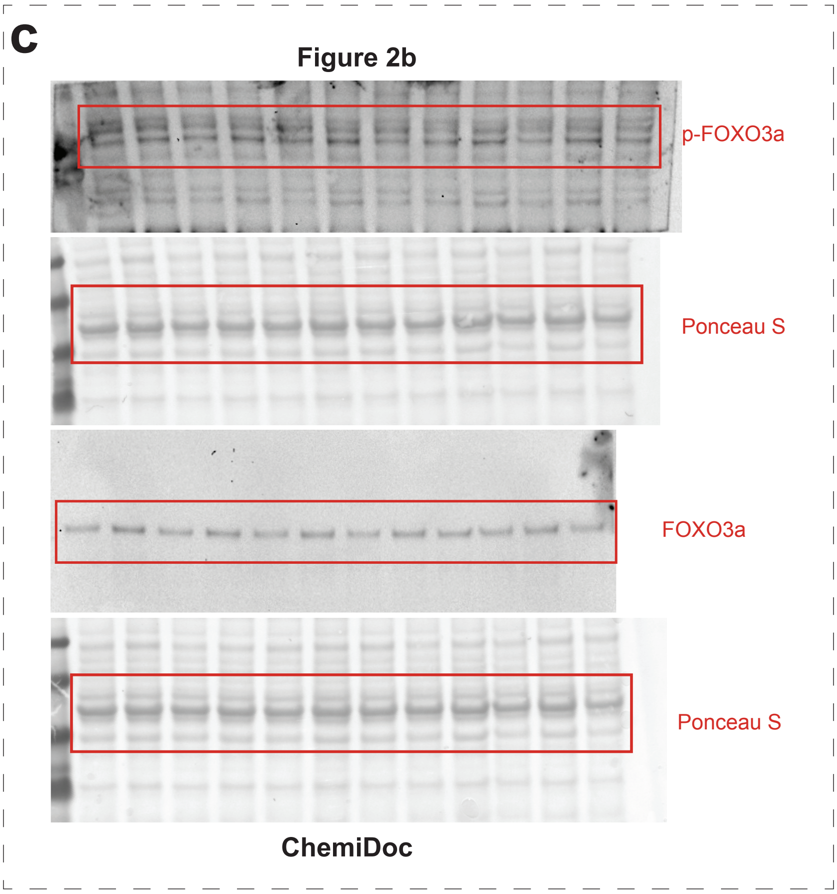

Supplementary Figure 2

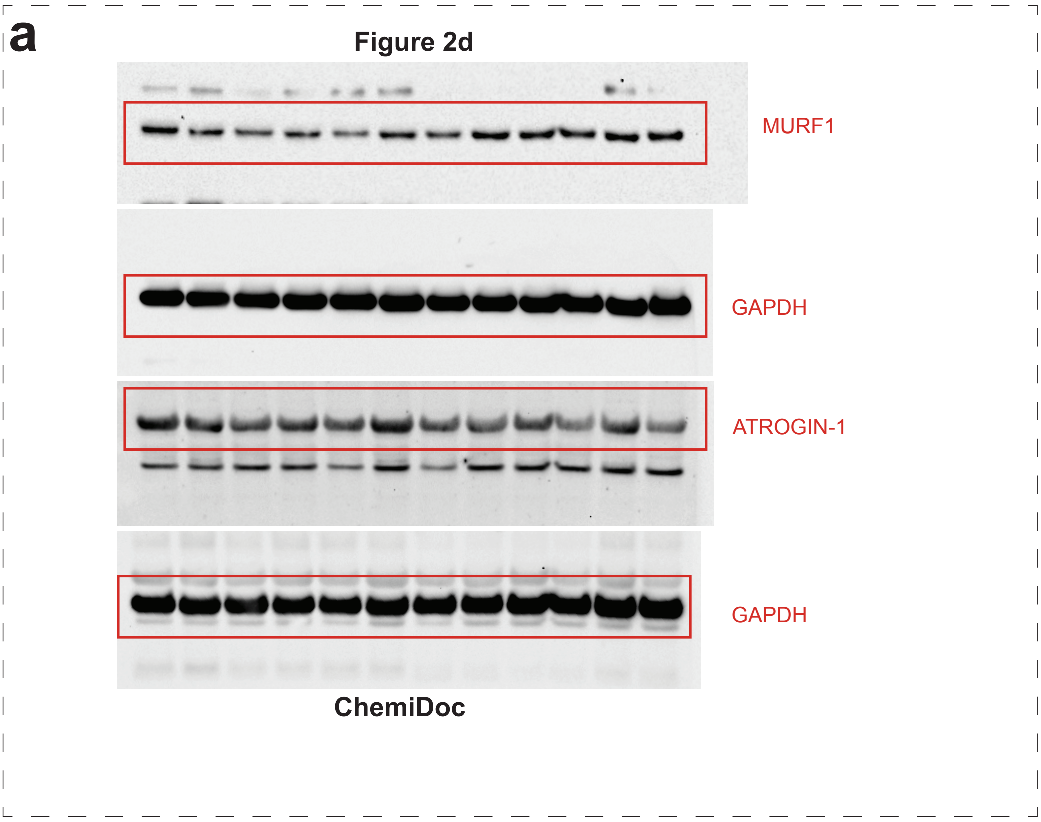

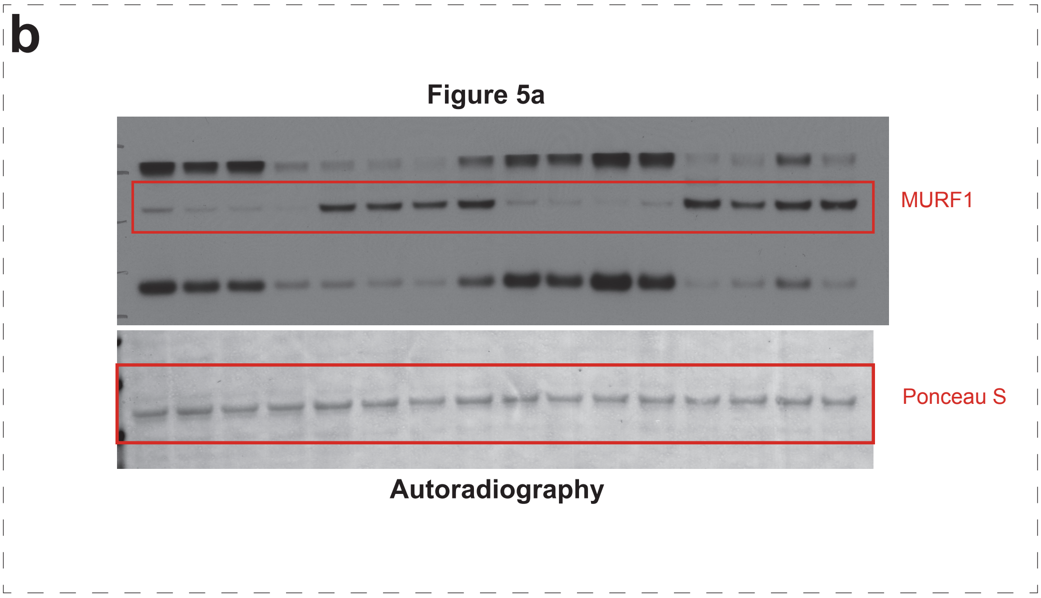

Supplement: Supplementary file 1 — Unprocessed western blot images [file 41531_2022_419_MOESM1_ESM.pdf]
