# Supplementary material for: Parkin deficiency exacerbates fasting-induced skeletal muscle wasting in mice
Source: NPJ Parkinsons Dis. 2022 Nov 17;8:159. doi: 10.1038/s41531-022-00419-3 (PMC9672397; doi:10.1038/s41531-022-00419-3)
Supplement: Supplementary file 3 — Reporting Summary [file 41531_2022_419_MOESM3_ESM.pdf]

nature research

Corresponding author(s): Ravi Kambadur

Reporting Summary

Nature Research wishes to improve the reproducibility of the work that we publish. This form provides structure for consistency and transparency in reporting. For further information on Nature Research policies, see Authors & Referees and the Editorial Policy Checklist.

Statistical parameters

When statistical analyses are reported, confirm that the following items are present in the relevant location (e.g. figure legend, table legend, main text, or Methods section).

n/a	Confirmed	
☐	☒	The exact sample size (n) for each experimental group/condition, given as a discrete number and unit of measurement
☐	☒	An indication of whether measurements were taken from distinct samples or whether the same sample was measured repeatedly
☐	☒	The statistical test(s) used AND whether they are one- or two-sided<br>*Only common tests should be described solely by name; describe more complex techniques in the Methods section.*
☒	☐	A description of all covariates tested
☐	☒	A description of any assumptions or corrections, such as tests of normality and adjustment for multiple comparisons
☐	☒	A full description of the statistics including central tendency (e.g. means) or other basic estimates (e.g. regression coefficient) AND variation (e.g. standard deviation) or associated estimates of uncertainty (e.g. confidence intervals)
☐	☒	For null hypothesis testing, the test statistic (e.g. F, t, r) with confidence intervals, effect sizes, degrees of freedom and P value noted<br>*Give P values as exact values whenever suitable.*
☒	☐	For Bayesian analysis, information on the choice of priors and Markov chain Monte Carlo settings
☒	☐	For hierarchical and complex designs, identification of the appropriate level for tests and full reporting of outcomes
☒	☐	Estimates of effect sizes (e.g. Cohen's d, Pearson's r), indicating how they were calculated
☐	☒	Clearly defined error bars<br>*State explicitly what error bars represent (e.g. SD, SE, CI)*

Our web collection on statistics for biologists may be useful.

Software and code

Policy information about availability of computer code

Data collection	, total RNA was isolated from 12-week-old Parkin KO and age-matched wild type Gas muscle using mirVANA™ miRNA isolation kit as per manufacturer's guidelines (Thermo Fisher Scientific). RNA integrity was assessed using Agilent RNA 6000 Nano Kit (Agilent Technologies, Santa Clara, CA, USA). Subsequent microarray analyses were performed by Molecular Genemics (Singapore) using Agilent SurePrint G3 Custom GE 8x60K, 1 color platform as following: 100ng/µl RNA was probed with Low Input AMP labelling Kit, One Color (Agilent p/n 5190-2305) as per manufacturer's instruction.
Data analysis	In vitro transcription of Pathway analysis was performed with significantly upregulated (>1.5 fold) and downregulated (<0.66 fold) genes with a p-value cut off <0.05 using Kyoto Encyclopedia of Genes and Genomes (KEGG) pathway database integrated in the Database for Annotation, Visualization and Integrated Discovery (DAVID) tool. Protein class analysis and identification of significantly upregulated genes was carried out using Protein Analysis Through Evolutionary Relationships (PANTHER) gene list analysis software.

For manuscripts utilizing custom algorithms or software that are central to the research but not yet described in published literature, software must be made available to editors/reviewers upon request. We strongly encourage code deposition in a community repository (e.g. GitHub). See the Nature Research guidelines for submitting code & software for further information.

Data

Policy information about availability of data

All manuscripts must include a data availability statement. This statement should provide the following information, where applicable:

- Accession codes, unique identifiers, or web links for publicly available datasets
- A list of figures that have associated raw data
- A description of any restrictions on data availability

Unprocessed western blot images were provided in Supplementary Material. Microarray data corresponding to Fed and Starved is available as Supplementary Data, and also at the ArrayExpress Archive of Functional Genomics Data, with the ArrayExpress accession number E-MTAB-12273 [https://www.ebi.ac.uk/biostudies/studies/E-MTAB-12273]. The other data that support the findings of this study are available from the corresponding author upon reasonable request.

Field-specific reporting

Please select the best fit for your research. If you are not sure, read the appropriate sections before making your selection.

☒ Life sciences ☐ Behavioural & social sciences

For a reference copy of the document with all sections, see nature.com/authors/policies/ReportingSummary-flat.pdf

Life sciences

Study design

All studies must disclose on these points even when the disclosure is negative.

Sample size	Western blot and qPCR analyses were performed using results of three independent experiments. For the analyses of WT and PARKIN KO mice, 4 mice in each group were sacrificed. 4 mice have been accepted by IACUC under the regulation of 3Rs.
Data exclusions	No was excluded except 1) EDL muscle weight data obtained from WT fasted mouse EDL, that has been damaged during dissection of the muscle tissue
Replication	All attempts at replication was successful and represented as they are.
Randomization	Allocations were random.
Blinding	Blinding was not possible as mice were dissected following starvation

Materials & experimental systems

Policy information about availability of materials

n/a	Involved in the study
☒	☐ Unique materials
☐	☒ Antibodies
☐	☒ Eukaryotic cell lines
☐	☒ Research animals
☒	☐ Human research participants

Antibodies

Antibodies used	Antibodies used in this study are as follows; ATROGIN1 (1:500, CST, PAB15627), MURF1 (1:500, Regeneron Pharmaceuticals), p-FOXO1 (1:1000, CST, 9461), FOXO1 (1:1000, CST, 2880), p-FOXO3 (1:1000, CST, 9466), FOXO3 (1:500, CST, 7497), GAPDH (1:10000, Santa Cruz, sc-32233), anti-mouse HRP (1:5000, Bio-Rad, 1706516), anti-rabbit HRP (1:5000, Bio-Rad, 1706515), anti-goat HRP (1:2000, Bio-Rad, 1721034)
Validation	Validations have been performed in our lab previously and given that the antibodies were commonly used in muscle research.

Eukaryotic cell lines

Policy information about cell lines

Cell line source(s)	C2C12 cells were purchased from ATCC
Authentication	None of the cell lines were authenticated
Mycoplasma contamination	C2C12 cells lines were tested negative for the mycoplasma

Commonly misidentified lines<br>(See ICLAC register)	N/A

Research animals

Policy information about studies involving animals; ARRIVE guidelines recommended for reporting animal research

Animals/animal-derived materials	Parkin knock out (Park2tm1Shn) mice with C57BL/6 background and wild type C57BL/6 mice were obtained from The Jackson Laboratory (Sacramento, CA, USA) and Biological Resource Centre (BRC, Singapore) respectively.

Method-specific reporting

n/a	Involved in the study
☐	☒ ChIP-seq
☒	☐ Flow cytometry
☒	☐ Magnetic resonance imaging

ChIP-seq

Data deposition

☒ Confirm that both raw and final processed data have been deposited in a public database such as GEO.

☒ Confirm that you have deposited or provided access to graph files (e.g. BED files) for the called peaks.

Data access links<br>*May remain private before publication.*	https://www.ebi.ac.uk/biostudies/studies/E-MTAB-12273
Files in database submission	E-MTAB-12273.idf, E-MTAB-12273.sdrf, KO690, KO691, KO692, KO693, KO697, KO698, KO699, KO700, WT1, WT2, WT3, WT4, WT5, WT6, WT7, WT8. As repository is dynamic, file names and types may be updated during the revision process.
Genome browser session<br>(e.g. UCSC)	no longer applicable

Methodology

Replicates	Gas muscles obtained from 4 different mouse for each control group WT Fed , WT Fasted, PARKIN KO Fed and PARKIN KO Fasted
Sequencing depth	In this manuscript, the microarray type used is a slide having 8 arrays with 60 thousand features per array. The exact number of features in the each array, inclusive of control probes, is 55,681 features. The microarray has a specific Agilent identifier called an AMADID which is used to identify what kind of array is being used; in this experiment the AMADID used is the design ID 028005.
Antibodies	N/A
Peak calling parameters	In this experiment setup, comparison is made between the 2 conditions as laid out below. Significance analysis is performed with T-test and Benjamini-Horchberg False Discovery Rate (FDR; q-value) and fold change analysis. The top 20 significant genes filtered on q-value are presented below. For the full list of genes, please refer to the Appendix material, Significant Genelist.
Data quality	In this analysis, a quality control (QC) on probeset is done via the information from the scanner QC check and summarized by the flag as laid out above.<br><br>Flags are categorical indicator from the scanner which indicates to user which probes the scanner has found to be erroneous. Probes flagged as “Not Detected” and “Compromised”, which typically are features that are detected to be non-uniform outlier features or not positive and significant etc (please refer to the picture below for the list of items that are flagged as “Not Detected” and “Compromised”).<br>Entities where at least 100.0 percent of samples in any 1 out of 2 conditions have flags in [Detected].
Software	The data analysis is done using a microarray specialized analysis software, Qlucore omics. User can download the software at website: http://www.qlucore.com/
